# A Two-Stage Bedside Intubation Method to Improve Success Rate of Post-pyloric Placement of Spiral Nasoenteric Tubes in Critically Ill Patients: A Multi-Center, Prospective Study

**DOI:** 10.3389/fmed.2022.875298

**Published:** 2022-05-12

**Authors:** Jing Xu, Sinian Li, Xiangyin Chen, Bo Tan, Shenglong Chen, Bei Hu, Zhiqiang Nie, Heng Ye, Cheng Sun, Ruibin Chi, Chunbo Chen

**Affiliations:** ^1^Department of Intensive Care Unit of Cardiovascular Surgery, Guangdong Cardiovascular Institute, Guangdong Provincial People's Hospital, Guangdong Academy of Medical Sciences, Guangzhou, China; ^2^Department of Critical Care Medicine, Guangdong Provincial People's Hospital, Guangdong Academy of Medical Sciences, Guangzhou, China; ^3^The Second School of Clinical Medicine, Southern Medical University, Guangzhou, China; ^4^Neurological Intensive Care Unit, Maoming People's Hospital, Maoming, China; ^5^Surgical Intensive Care Unit, Maoming People's Hospital, Maoming, China; ^6^Department of Emergency, Maoming People's Hospital, Maoming, China; ^7^Department of Cardiology, Guangdong Cardiovascular Institute, Guangdong Provincial People's Hospital, Guangdong Academy of Medical Sciences, Guangzhou, China; ^8^Department of Critical Care Medicine, Guangzhou First People's Hospital, School of Medicine, South China University of Technology, Guangzhou, China; ^9^Department of Critical Care Medicine, Xiaolan People's Hospital of Zhongshan, Zhongshan, China; ^10^Department of Critical Care Medicine, Maoming People's Hospital, Maoming, China

**Keywords:** self-propelled nasoenteric tubes, prokinetic agents, blind bedside, post-pyloric placement, enteral nutrition, critically ill patients

## Abstract

**Backgrounds:**

Prokinetic agents could improve the success rate of post-pyloric placement of self-propelled spiral nasoenteric tubes (NETs), and bedside blind technique might apply as a rescue therapy subsequent to spontaneous transpyloric migration failure. The objective of this study was to investigated the validity and safety of these two bedside intubation methods as a sequential procedure for post-pyloric placement of spiral NETs in critically ill patients.

**Methods:**

The multicenter, prospective study was conducted in intensive care units of four tertiary hospitals (June 2020 to January 2021). Eligible patients received self-propelled spiral NET placements, promoted by prokinetic agents (Stage 1). An abdominal X-ray performed 24 h post-intubation confirmed the position of the tube tip. Patients with a failed transpyloric migration entered Stage 2, where beside blind intubation was conducted (reconfirmed by X-ray). The primary end point was the overall success rate of post-pyloric placement.

**Results:**

The overall success rate of post-pyloric placement of the spiral NET was 91.1% (73.4% in the third portion of the duodenum [D3] or beyond). The total adverse event rate was 21.0%, without any serious adverse events. In Stage 1, 55.6% of participants achieved transpyloric migration, of these, 44.4% migrated to D3 or beyond. The median time from decision to intubate to the initiation of enteral nutrition (EN) was 25 h. In Stage 2, 83.0% of patients had successful post-pyloric intubation (67.9% in D3 or beyond). The median time from decision to EN initiation after the two-stage process was 36 h.

**Conclusions:**

Prokinetic agents-assisted self-propelled intubation and remedial bedside blind technique as a sequential procedure for post-pyloric placement of spiral NETs were effective and safe, and this two-stage process did not affect the implementation of early EN in critically ill patients.

**Trial Registration:**

Chinese Clinical Trial Registry, ChiCTR1900026381. Registered on 6 October 2019.

## Introduction

Clinical nutrition support is one of the basic elements for the comprehensive treatment of critically ill patients, and consists of providing the patient with all necessary nutrients for normal operation of the body (1). Major clinical practice guidelines agree that enteral nutrition (EN) via tube feeding is currently the preferred method to feed critically ill patients and it should be implemented within 48 h if oral intake is not possible (2–4). Evidence suggests that post-pyloric feeding, in which the nutrients are delivered directly into the duodenum or jejunum, could reduce the risk of aspiration pneumonia, increase the total nutrients delivered to the patient, and provide additional benefits over routine gastric administration (5–7). Unfortunately, placement of the post-pyloric feeding tube can be challenging and technically difficult. To date, a consensus has not been reached regarding an effective and preferred method for post-pyloric tube placement.

Conventional post-pyloric tube placement relies on endoscopic or radiographic technology and the patient is usually required to be transported from the ward to an endoscopy center or radiology department, which may exacerbate the patient's condition and delay early EN (8, 9). In recent years, ultrasonic- and electromagnetic-assisted intubation techniques have been applied in clinical practice, which are reliable but slightly expensive (10–13). In brief, the methods mentioned above are highly device-dependent and difficult to popularize. Certain studies have investigated an alternative method using self-propelled spiral nasoenteric tubes (NETs) for post-pyloric feeding of critically ill patients (14–18). However, despite the use of prokinetic agents, only half of these patients achieve post-pyloric feeding tube placement, which is completely inadequate to meet the clinical needs of severely ill patients (19–22). Another study showed a blind bedside technique applied as a rescue therapy after a failed spontaneous transpyloric migration in patients had a success rate up to 80% (23). Based on the above studies, it was speculated that combining these two non-device-dependent bedside intubation methods as a sequential procedure may yield an encouraging success rate of post-pyloric tube placement. However, this two-stage process may automatically increase 24-h EN initiation, and whether it will affect the implementation of early EN in critically ill patients remains to be confirmed by further studies.

Therefore, this multicenter, prospective study was designed to investigate the efficacy and safety of prokinetic agents-assisted self-propelled intubation and remedial bedside blind technique as a sequential procedure for post-pyloric placement of spiral NETs.

## Materials and Methods

### Study Design

This prospective, multicenter study was conducted in the intensive care units (ICUs) of four tertiary hospitals in China. The study protocol received approvals from China Ethics Committee of Registering Clinical Trials (approval number ChiECRCT20190167). Written informed consent forms were obtained from each patient or from the next of kin. The trial was registered with the Chinese Clinical Trial Registry Center (ChiCTR1900026381).

### Participants

Between June 2020 and January 2021, consecutive patients admitted to the ICU who were at least 18 years of age, required EN for more than 3 days, had increased gastric residues (single measurement >150 mL or 12 h cumulative volume > 500 mL) (24), and signed the written informed consent were recruited for the present study. Exclusion criteria were: any indication for percutaneous gastrostomy or jejunostomy; varices or stenosis of the esophagus, or if the patient had previously undergone major gastroesophageal surgery such as an esophagectomy or gastrectomy; active upper gastrointestinal bleeding; severe nasopharyngeal injury or stenosis; severe coagulation dysfunction; gastric malignancy, peptic ulcer, or mechanical ileus; pregnancy; contraindications of erythromycin or metoclopramide; and history of allergy to meglumine diatrizoate.

### Training Program

First, a 60-min training program, including a manual and video presentation of the study protocol and procedures for bedside blind placement of spiral NETs, was developed by a senior intensivist based on previous studies (19–21, 23, 25). Then, intensive training was given to all operators (ICU physicians and bedside nurses) involved in the study. Upon completion of the theoretical training, all operators were required to watch five tube placements and perform at least 5 procedures under the supervision of the above senior intensivist (26).

### Study Intervention

#### Stage 1: Self-Propelled Spiral NET Placement Facilitated by Prokinetic Agents

A long spiral NET (145 cm) made of radiopaque polyurethane, with an inner diameter of 2.4 mm and an outer diameter of 3.3 mm, and 2.5 rings with a diameter of 3 cm in the front segment (CH10, Flocare Bengmark, Nutricia, Wuxi, China) was used in present study.

In the first stage (Stage 1), all eligible patients underwent spontaneous transpyloric feeding tube placement with the use of prokinetic agents. According to the operating instructions, the feeding tube was inserted by a single operator when the patient was in the supine position. The tube and the stylet were wetted with saline or sterile water to activate the hydrophilic lubrication material that coated their surfaces. The operator completely inserted the stylet into the tube to straighten it, measured the initial insertion depth (approximately the distance from the xiphoid process to the nasal tip to the earlobe), and then gently inserted the tube along the wall of one nostril. As the tube was inserted into the larynx, the patient's head was bent slightly toward their chest and the tube was pushed slowly forward until the tip had reached the target position. Gas (20 mL) was injected through the tube to confirm that the tube tip had reached the stomach. The stylet was withdrawn approximately 25 cm with gentle tugs until loose, the tube was inserted 25 cm further, and finally the guide wire was completely removed. The tube was then fixed to the patient's face with a free loop of approximately 40 cm, and the distance of migration was observed. If the tube automatically moved down 25 cm, the tube end was considered to have reached the proximal jejunum, and the tube was then fixed on the patient's nose with tape. Otherwise, the observation continued for 24 h, at which time the tube was fixed on the patient's nose with tape and an abdominal X-ray examination was completed to confirm the position of the tube end.

According to the trial protocol, if the patient's weight was ≤ 40 kg, a metoclopramide injection (10 mg) was given intravenously every 12 h. If the patient's weight was > 40 kg, a metoclopramide injection (10 mg) was given intravenously every 8 h. In patients with renal insufficiency, the dose was halved. When patients had contraindications to metoclopramide, erythromycin (250 mg dissolved in 100 mL 0.9% saline) was administered intravenously every 6 h for a total of 1000 mg.

An abdominal X-ray was performed 24 h after initiation of the tube placement to confirm the location of the tube end. If the tube tip was located beyond the pylorus, the post-pyloric placement in Stage 1 was considered successful and EN was initiated. If the tube end was still in the stomach, the spontaneous transpyloric migration was considered to have failed, and the patient proceeded to Stage 2 of the intervention.

#### Stage 2: Remedial Bedside Blind Technique for Post-pyloric Placement of Spiral NETs

If the spontaneous transpyloric migration failed, the patient was reevaluated for blind bedside post-pyloric placement. As described in previous study, the remedial technique consists of three phases (esophageal, intragastric, and post-pyloric placement), which can be achieved with specific manipulation in minutes, and the tube was inserted to a depth of approximately 100 cm. During the operation, it was critical to determine the position of the tube tip at each phase before proceeding to the next phase, which is one of the keys to ensuring the success of this technique.

In preparation, the patient was placed in the supine position without raising the head of the bed. The spiral tube, if it was not damaged during Stage 1, was washed with normal saline and fully lubricated with paraffin after removal for further use. Patients were given an intravenous bolus of metoclopramide (20 mg) 10 min before tube placement. The dose was halved in patients with renal insufficiency, or not used if contraindicated. The initial insertion depth was measured using the same method described in Stage 1, and then insertion was started.

##### Esophagus and Gastric Placement

The operator tilted the patient's head back and first inserted the tube through one nostril, then bent the patient's head forward and gently pushed the tube into esophagus. If the patient was conscious, they were asked to cooperate by swallowing when the tube reached the pharynx. The key to this phase was to push the tube into the esophagus through the epiglottis rather than into the trachea through the glottis. The tube was then inserted forward to the measured distance before intubation. A combination of air injection and upper abdominal auscultation (The whoosh test) was performed to determine the position of the tube tip, and a gurgling was regarded as a sign that the tube had been inserted into the stomach (27). If it was uncertain whether the tube has been inserted in the stomach, the tube was withdrawn to the nasal cavity and re-inserted.

##### Post-pyloric Placement

Once it was confirmed that the tube has been inserted into the stomach. The head of the bed was raised approximately 30 degrees. Air (100 mL) was injected at each 5 cm interval and pumped back with a syringe until the gastric cavity surrounding the tube expanded or the pylorus opened, which allowed the tube to pass through the pylorus successfully. When 100 mL of air was injected and less than 20ml was pumped back, it indicated that the tube end has passed the pylorus (28). Finally, the tube was inserted about 100 cm, with the tube end reaching or beyond the duodenum (or jejunum). At this phase, the whoosh test was also used to determine the position of the tube end, and if a gurgling was heard loudest in the midline of the epigastrium, it indicated that the end has reached the gastric antrum. Once the tube tip was inserted through the pylorus into the duodenum, the loudest gurgling could be heard in the right upper abdomen (27). PH test was performed on all the digestive juices pumped back (when pH <5, it indicated gastric juice; when pH > 7, it indicated small intestine juice) (29). The guide wire withdrawal test was evaluated the coiling of the tube in the stomach (25). After ensuring the tip of the tube had passed through the pylorus, the guide wire was extracted and normal saline (20–30 mL) was injected into the tube as a rinse. Finally, the tube was fixed to the patient's nose with adhesive tape.

The position of the tube was confirmed by an additional abdominal X-ray. When the tube end was determined to be passed the pyloric sphincter, the second stage was considered successful and EN was started. If the position of the tube was still not appropriate, other methods of post-pyloric feeding tube placement were selected according to the patient's condition.

### Data Collection

Once patients were enrolled, their baseline data including demographic characteristics, preliminary diagnosis, concomitant medication, and mechanical ventilation status were collected from their medical records. The severity of illness as assessed by the Acute Physiology and Chronic Health Evaluation II (APACHE II) score, Sequential Organ Failure Assessment (SOFA) score and Acute Gastrointestinal Injury (AGI) grade were also recorded.

The following variables were recorded during both stages of intubation: time to insertion, number of attempts, success rate, and the time from decision to intubate to the initiation of EN (the time from decision to EN initiation). The position of the tube end, as determined by X-ray, was documented as either the stomach, the first, second, third, or forth portion of the duodenum (D1–D4, respectively), or the proximal jejunum.

Adverse events related to either the prokinetic agents or the tubes were recorded over the course of the study. Vital signs including the heart rate (HR), respiratory rate (RR), mean arterial pressure (MAP), and pulse oxygen saturation (SpO_2_) were monitored and transcribed every 5 min, beginning at the start of tube insertion, and ending 30 min after tube insertion.

### Study Endpoints

The primary endpoint was the overall success rate of post-pyloric placement of the spiral NET (ie: the tube tip had reached the first portion of the duodenum or beyond, as confirmed by X-ray 24 h after insertion). The secondary endpoints included the total proportion of the tube end passing through the third portion of the duodenum (D3) or beyond, and the time from decision to EN initiation in patients with a successful post-pyloric placement in either Stage 1 or 2. Additional study endpoints included the incidence of adverse events caused by the prokinetic agents or the tubes, as well as changes in HR, RR, MAP and SpO_2_ of patients prior to, at the end of, and 30 min after tube insertion.

### Statistical Analysis

Previous studies have shown that the success rate of post-pyloric placement of spiral NETs assisted by prokinetic agents (metoclopramide or erythromycin) was 55–57.5% (19, 20), and the success rate of bedside blind intubation was approximately 81.9% for patients with transpyloric migration failure (23). Based on these findings, it was estimated that the combination of these two bedside intubation methods as a sequential procedure would lead to successful post-pyloric placement in more than 88% of patients. A sample size of 112 patients would yield 80% power to detect a 5% reduction between the null hypothesis proportion of 88%, and an exact binomial hypothesis test with a target significance level of 0.05. The sample size was adjusted by 10% to allow for ineligible patients and those lost to follow-up. Therefore, the final sample size chosen for this study was 124 patients.

The Shapiro–Wilk test was used to detect the normality of data distributions. Quantitative variables were presented as a mean ± standard deviation or as median (interquartile ranges, IQR) according to the distribution, and as occurrences (percentage) for qualitative variables. Vital signs were compared by paired *t*-tests or non-parametric tests according to the normality of variables. All statistical analyses were performed with SPSS 21.0 (SPSS Inc., Chicago, IL, USA). A two-sided *P*-value < 0.05 was considered statistically significant.

## Results

### Demographics and Baseline Characteristics

**Figure 1:**
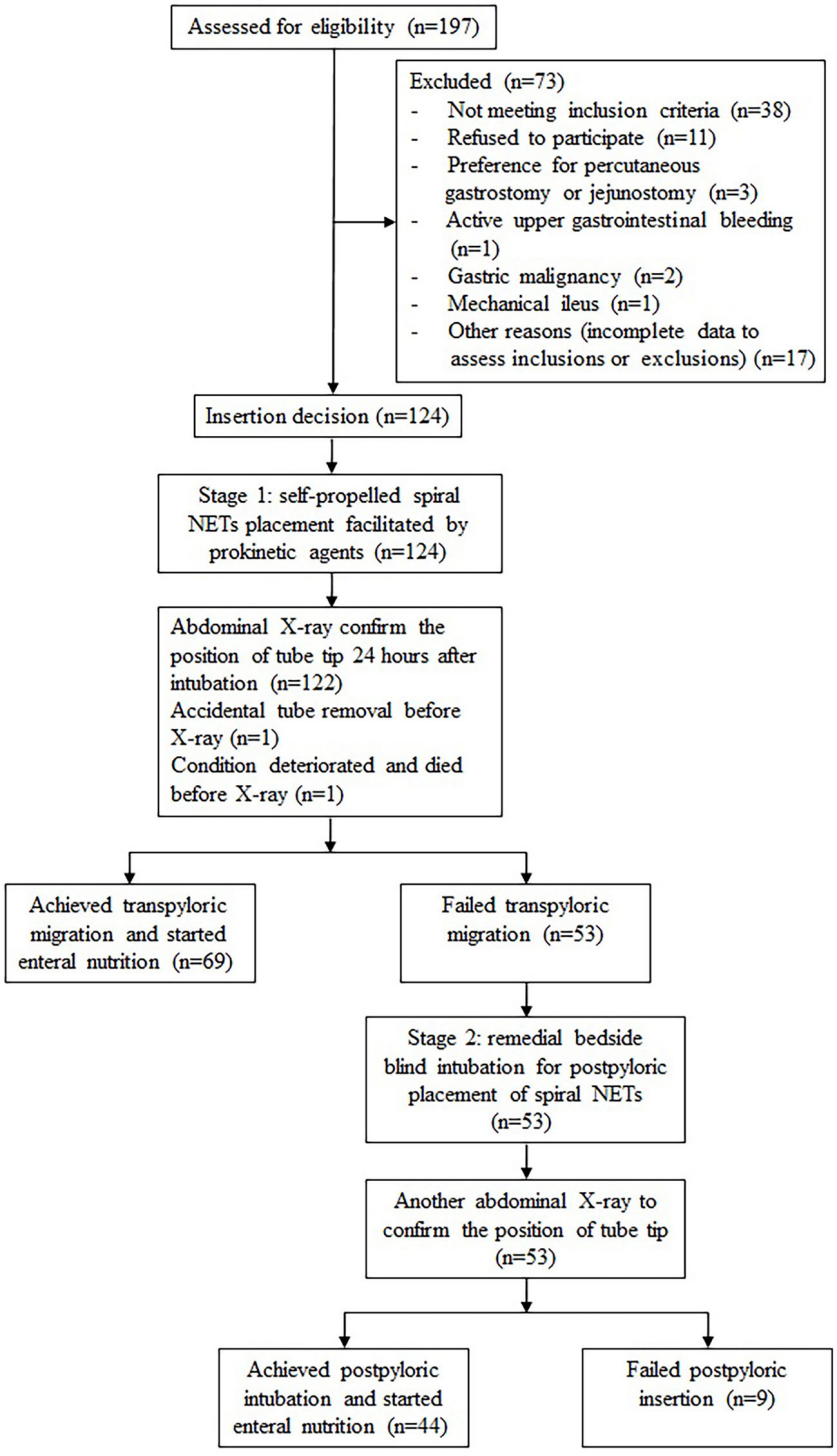
Flowchart of the study population, NETs nasoenteric tubes.

Of 197 patients who were assessed for eligibility, 124 were enrolled in stage 1, but the intervention was discontinued prior to abdominal X-ray in 2 patients, and 53 patients proceed to stage 2 (Figure 1). Patients in both stages were predominantly male over 60 years of age, neurological and respiratory diseases were the most common primary diagnoses, and more than 80% of the patients were treated with mechanical ventilation. The proportion of sedatives and analgesics increased in stage 2. The patients with AGI grade II were the most, accounting for 54.9% in stage 1 and 60.4% in stage 2 (Table 1).

**Table 1:** Patient demographics and baseline clinical characteristics.

**Variables**	**Stage 1 (*n* = 124)**	**Stage 2 (*n* = 53)**
Age, years	63 (53–74)	62 (54–71)
Gender, male	80 (64.5)	36 (67.9)
BMI, kg/m^2^	22.5 (20–25)	23 (20–25)
Hypertension	35 (28.2)	16 (30.2)
Diabetes	18 (14.5)	6 (11.3)
Primary diagnosis		
Neurological	55 (44.4)	23 (43.4)
Respiratory	37 (29.8)	20 (37.7)
Cardiovascular	3 (2.4)	2 (3.8)
Gastrointestinal	4 (3.2)	3 (5.6)
Multiple trauma	15 (12.1)	2 (3.8)
Sepsis	2 (1.6)	1 (1.9)
Others	8 (6.5)	2 (3.8)
Use of sedatives	60 (48.4)	28 (52.8)
Use of analgesics	47 (37.9)	22 (41.5)
Use of vasopressors	20 (16.1)	9 (17.0)
Mechanical ventilation	101 (81.4)	44 (83.0)
APACHE II score	19 (16–23)	20 (18–24)
SOFA score	7 (5–9)	8 (5–10)
AGI grade		
Without AGI	7 (5.6)	2 (3.8)
I	38 (30.6)	13 (24.5)
II	68 (54.9)	32 (60.4)
III	11 (8.9)	6 (11.3)

*Quantitative variables were presented as median (interquartile range, IQR) and qualitative variables as number (percentage, %)*.

*BMI, body mass index; APACHE II, acute physiology and chronic health evaluation II; SOFA, sequential organ failure assessment; AGI, acute gastrointestinal injury*.

### Primary Endpoint and Secondary Endpoints

The overall success rate of the post-pyloric placement of the spiral tube was 91.1% (113/124) and the total proportion placed in D3 or beyond was 73.4% (91/124). In Stage 1, 69 of 124 participants (55.6%) achieved transpyloric migration. In 55 of these patients (44.4%), the tube had migrated to D3 or beyond. The median time of tube insertion was 10 min, with an average of 1.3 attempts. The median time from decision to EN initiation was 25 h (IQR 23–27). In Stage 2, 44 of the 53 patients (83.0%) had successful post-pyloric intubation, and 36 (67.9%) had tube placement in D3 or beyond. The procedure lasted a median time of 13 min, and the average number of attempts was 1.6. The median time from decision to EN initiation after the two-stage protocol was 36 h (IQR 30.5–45) (Table 2).

**Table 2:** Primary and secondary endpoints.

**Endpoints**	**Value in total study sample (*n* = 124)**
Primary endpoint	
Post-pyloric placement^a^	113 (91.1)
Secondary endpoints	
Placed at D3^b^ or beyond	91 (73.4)
Time 1^c^, h	25 (23–27)
Time 2^d^, h	36 (30.5–45)

*Quantitative variables were presented as median (interquartile range, IQR) and qualitative variables as number (percentage, %)*.

a *Post-pyloric placement, reaching the first portion of the duodenum or beyond*.

b *D3, the third portion of the duodenum*.

c *Time 1, the time from decision to intubate to initiation of enteral nutrition for successful patients in stage 1*.

d *Time 2, the time from decision to intubate to initiation of enteral nutrition for successful patients after two-stage process*.

### Safety Endpoints

The total adverse event incidence was 21.0%. Of these, 14.5% occurring in Stage 1, and 18.9% occurred in Stage 2. Importantly, no serious adverse events were observed (Table 3). Nausea, pain, and nasal mucosal bleeding were the most common tube-related adverse events in both stages of intubation. Adverse reactions to the prokinetic agents were rare, and it is worth noting that these adverse events were alleviated spontaneously without additional medication or intervention.

**Table 3:** Adverse events.

**Event**	**Stage 1** **(*n* = 124)**	**Stage 2** **(*n* = 53)**	**Total** **(*n* = 124)**
Any event	18 (14.5)	10 (18.9)	26 (21.0)
Tube-associated events	14 (11.3)	8 (15.1)	21 (16.9)
Nasal mucosa bleeding	4 (3.2)	2 (3.8)	6 (4.8)
Airway misplacement	1 (0.8)	1 (1.9)	2 (1.6)
Pain	3 (2.4)	2 (3.8)	5 (4.0)
Nausea	7 (5.6)	5 (9.4)	12 (9.7)
Vomiting	2 (1.6)	2 (3.8)	4 (3.2)
Prokinetic agents-associated events	5 (4.0)	2 (3.8)	6 (4.8)
Rash	1 (0.8)	0 (0)	1 (0.8)
Amyostasia	1 (0.8)	1 (1.9)	2 (1.6)
Lethargy	1 (0.8)	0 (0)	1 (0.8)
Dysphoria	2 (1.6)	1 (1.9)	2 (1.6)

*Quantitative variables were presented as number (percentage, %)*.

Vital signs were monitored and transcribed during both stages of tube placement (Table 4). The HR, RR and MAP were slightly increased compared to the monitored vital signs before and at the end of tube insertion, while SpO_2_ remained relatively stable. However, there was no statistical difference in vital signs assessed before and 30 min after tube placement (P > 0.05)

**Table 4:** Vital signs monitored peri-procedure.

**Vital signs**	**Pre- procedure**	**End of-** **procedure**	**Post- procedure^a^**	***P*** **-value**	
				**Pre-procedure vs. End of-procedure**	**Pre-procedure vs. Post-procedure**
Stage 1					
HR, bpm	88.6 ± 15.2	93.3 ± 16.0	89.3 ± 14.8	<0.0001	0.1163
RR, rpm	18.4 ± 3.9	19.5 ± 4.6	18.5 ± 4.0	<0.0001	0.2792
MAP, mmHg	90.2 ± 10.8	93.6 ± 12.3	90.6 ± 10.6	<0.0001	0.1326
SpO_2_, %	98.7 ± 1.8	98.6 ± 1.8	98.7 ± 1.8	0.1963	0.7857
Stage 2					
HR, bpm	90.7 ± 16.4	97.2 ± 16.9	92.0 ± 16.5	<0.0001	0.0942
RR, rpm	19.1 ± 4.0	20.4 ± 4.5	19.3 ± 4.1	<0.0001	0.2066
MAP, mmHg	91.2 ± 11.2	96.4 ± 11.6	92.3 ± 10.1	<0.0001	0.1815
SpO_2_	98.6 ± 1.7	98.4 ± 2.0	98.5 ± 2.0	0.0802	0.5681

*Quantitative variables were presented as mean ± standard deviation*.

*HR heart rate, RR respiratory rate, MAP mean arterial pressure, SPO_2_ pulse oxygen saturation, bpm beats per minute, rpm respirations per minute, mmHg millimeters of mercury*.

a *Post-procedure, data were collected 30 min after tube insertion*.

## Discussion

This prospective, multicenter study revealed that prokinetic agents-assisted self-propelled intubation and remedial bedside blind technique as a sequential process for post-pyloric placement of spiral NETs were effective and safe in critically ill patients. The overall success rate of post-pyloric placement was 91.1%, which was an encouraging result and confirmed our hypothesis. In addition, the median time from decision to EN initiation was 36 h after the two-stage intubation process, which meets the guideline recommended golden window (48 h) for early EN (2–4). The incidence of adverse events observed over the course of the study was 21.0%, and there were no serious adverse events observed. Therefore, it is believed that the clinical application of this two-stage intubation strategy is promising, and will allow more patients in the ICU setting to benefit from early EN and avoid more invasive fluoroscopic or endoscopic tube insertion protocols.

Enteral tube feeding is an effective method to provide nutritional support to patients who have functional intestinal tracts but may not be able to meet their nutritional needs via a standard oral diet (30). Early nutritional support can not only maintain the integrity of intestinal structure and function, promote gut-mediated immunity, and enhance the body's resistance to severe diseases, but also reduces pneumonia caused by reflux and aspiration, and can even improve the clinical outcome in patients suffering from severe conditions (31–33). In the current prospective study, nearly half of the participants had a preliminary diagnosis of neurological disorders, such as cerebral hemorrhage and cerebral infarction, which could result in dysphagia and render the oral intake of food impossible. In addition, more than 80% of participants were being treated with mechanical ventilation, which also results in the inability to consume food orally and the need for EN. However, in a large collaborative cohort study, only 5.5% of patients in ICUs were able to receive nasoenteric nutritional support (34). An important reason for this low number may be the lack of an effective method of transpyloric tube placement.

Although various techniques have been used clinically to place post-pyloric feeding tubes, no unified guidelines exist for these methods. Device-assisted post-pyloric tube placement techniques, such as endoscopic, fluoroscopic, electromagnetic, and ultrasonic guidance, are highly device-dependent and greatly influenced by specialized equipment and operator proficiency (8–13). Therefore, these methods have been difficult to popularize, especially in areas with limited medical resources. Non-device-assisted bedside post-pyloric placement methods, including spontaneous passage with or without motility agents (17–20) and several blind beside techniques (23, 35–37), may be a convenient and viable option. However, the overall success rate of bedside intubation has been relatively low, but is worthy of additional study to improve the efficiency of transpyloric placement.

In an effort to improve the current situation, our team has conducted a series of studies regarding the optimal placement of spiral NETs, and based on these previous studies, we designed this two-stage bedside intubation study, in which self-propelled process was attempted as the first step and blind bedside technique as the second step (19–23). As a newly introduced technique, blind bedside post-pyloric placement is not easy, particularly in the critical illness setting, and this technique is not routinely taught in current ICU training programs in mainland China, resulting in a lack of relevant experience for intensive care physicians (38). In addition, blind bedside intubation technique, in which the feeding tube is inserted directly into the small intestine, is completely unguided. Although the success rate can reach more than 80%, it may lead to serious complications, such as pneumothorax and gastric perforation (39–41). In contrast, self-propelled NET placement procedure is the same as the indwelling of a gastric tube, which is a routine clinical training skill. Moreover, in previous studies, we observed that more than half of patients achieved transpyloric migration assisted by prokinetic drugs, and the adverse events were mild (19–21). Therefore, based on the current situation of ICUs in Our country, we believed that it is significant to choose a relatively safe and easy to operate bedside intubation method as the first option, rather than directly choosing bedside blind technique, despite its relatively high success rate. Importantly, our study achieved an overall success rate of 91.1% for post-pyloric placement, which was comparable to device-dependent assisted techniques (85–93%) (42).

In addition, studies have shown that early EN (within 48 h) was effective in reducing the incidence of pulmonary infection, promoting nutrition status, enhancing early recovery of intestinal motility, and reducing the length of hospital stay and hospital costs (43–45). In this study, we recorded the time from decision to EN initiation to determine whether the implementation of early EN was delayed in critically ill patients due to the 24-h waiting period or the two-stage process. Ultimately, we observed that the median time from decision to EN initiation was 25 h (stage 1) and 36 h (two-stage process), which did not exceed the guideline recommended optimal implementation time of EN (48 h) (2–4). Therefore, we believed that it is meaningful to combine these two bedside intubation methods as a sequential process for post-pyloric placement of spiral NETs in the current study.

More importantly, the total incidence of adverse events was 21.0%. No serious adverse events were observed in this two-stage study, such as prolonged hospitalization, cause disability, endanger life or death. On the one hand, all the operators, whether ICU physicians or bedside nurses, were medical staff from tertiary hospitals in China. They were all very professional and had extensive clinical experience in ICUs. On the other hand, prior to the implementation of this study, training program was conducted for all the operators, including a 60-min theoretical training and operational training program of intubation procedures. In addition, previous studies have found that significant improvements in the effectiveness of all trained operators and a significant reduction in adverse events among primary intensivists as increased operating experience (26). In general, operator experience played a significant role in the success and safety of post-pyloric tube placement, and a rigorous intensive training program ensured the implementation of our study.

However, this study does have certain limitations. First, this study did not have a randomized controlled design and lacked a control group or direct comparison with device- dependent feeding tube placement techniques. Second, the sample size of this study was small, which may limit the evaluation of tube-related or prokinetic agents-associated adverse events. Third, the cost of placing a post-pyloric feeding tube was not evaluated in this study, making it impossible to evaluate the health economics of this two-stage intubation strategy. Therefore, further studies are needed for a comprehensive evaluation to promote the application of non-device-dependent bedside intubation technology.

## Conclusion

Prokinetic agents-assisted self-propelled intubation and remedial bedside blind technique as a sequential procedure for post-pyloric placement of spiral NETs were effective and safe, and this two-stage process did not affect the implementation of early EN in critically ill patients.

## Data Availability Statement

The raw data supporting the conclusions of this article will be made available by the authors, without undue reservation.

## Ethics Statement

The studies involving human participants were reviewed and approved by the ethical standards of China Ethics Committee of Registering Clinical Trials (approval number ChiECRCT20190167). The patients/participants provided their written informed consent to participate in this study. Written informed consent was obtained from the individual(s) for the publication of any potentially identifiable images or data included in this article.

## Author Contributions

JX, BH, ZN, and CC contributed to the conception and the design of the study and interpretation of the data and critically revised the manuscript. JX, SL, XC, HY, CS, and RC performed the study and collected data. JX, BT, and SC analyzed the data. All authors contributed to the acquisition and analysis of the data, drafted the manuscript, agree to be fully accountable for ensuring the integrity and accuracy of the work. All authors read and approved the final manuscript.

## Funding

CC is currently receiving a grant (Grant no. DFJH2020028) from the Major Program of Summit Project, Guangdong Province High-level Hospital Construction Project of Guangdong Provincial People's Hospital, Guangdong Academy of Medical Sciences. SC is currently receiving a grant (Grant no. A2020540) from Guangdong Medical Scientific Research Foundation.

## Conflict of Interest

The authors declare that the research was conducted in the absence of any commercial or financial relationships that could be construed as a potential conflict of interest.

## Publisher's Note

All claims expressed in this article are solely those of the authors and do not necessarily represent those of their affiliated organizations, or those of the publisher, the editors and the reviewers. Any product that may be evaluated in this article, or claim that may be made by its manufacturer, is not guaranteed or endorsed by the publisher.

## Abbreviations

NETs: Nasoenteric tubes
EN: Enteral nutrition
ICUs: Intensive care units
D3: third section of the duodenum
APACHE II: Acute Physiology and Chronic Health Evaluation II
SOFA: Sequential Organ Failure Assessment
AGI: Acute gastrointestinal injury
IQR: Interquartile range
SD: Standard deviation
BMI: Body mass index
HR: Heart rate
RR: Respiratory rate
MAP: Mean arterial pressure
SpO2: Pulse oxygen saturation.
